# *Plasmodium vivax* Malaria Relapse Risk Depends on Transmission Intensity: Evidence From a Longitudinal Study in Northwest Thailand

**DOI:** 10.1093/ofid/ofaf667

**Published:** 2025-10-30

**Authors:** Cindy S Chu, Khin Maung Lwin, Kathy Burgoine, Aung Pyae Phyo, Claudia Turner, Thida San, Aye Aye Aung, Htun Htun Win, Klay Htoo, Nay Lin Soe, Naw Christina, Hla Hla Than, Naw Dah, Kasiha Pilaseng, Jacher Wiladpaingern, Stephane Proux, Mavuto Mukaka, Daniel M Parker, Verena I Carrara, François Nosten, Nicholas J White

**Affiliations:** Shoklo Malaria Research Unit, Mahidol Oxford Tropical Medicine Research Unit, Mae Sot, Thailand; Lao-Oxford-Mahosot Hospital–Wellcome Trust Research Unit, Mahidol Oxford Tropical Medicine Research Unit, Vientiane, Lao People's Democratic Republic; Centre for Tropical Medicine and Global Health, Nuffield Department of Medicine, University of Oxford, Oxford, United Kingdom; Shoklo Malaria Research Unit, Mahidol Oxford Tropical Medicine Research Unit, Mae Sot, Thailand; Shoklo Malaria Research Unit, Mahidol Oxford Tropical Medicine Research Unit, Mae Sot, Thailand; Shoklo Malaria Research Unit, Mahidol Oxford Tropical Medicine Research Unit, Mae Sot, Thailand; Shoklo Malaria Research Unit, Mahidol Oxford Tropical Medicine Research Unit, Mae Sot, Thailand; Centre for Tropical Medicine and Global Health, Nuffield Department of Medicine, University of Oxford, Oxford, United Kingdom; Shoklo Malaria Research Unit, Mahidol Oxford Tropical Medicine Research Unit, Mae Sot, Thailand; Shoklo Malaria Research Unit, Mahidol Oxford Tropical Medicine Research Unit, Mae Sot, Thailand; Shoklo Malaria Research Unit, Mahidol Oxford Tropical Medicine Research Unit, Mae Sot, Thailand; Shoklo Malaria Research Unit, Mahidol Oxford Tropical Medicine Research Unit, Mae Sot, Thailand; Shoklo Malaria Research Unit, Mahidol Oxford Tropical Medicine Research Unit, Mae Sot, Thailand; Shoklo Malaria Research Unit, Mahidol Oxford Tropical Medicine Research Unit, Mae Sot, Thailand; Shoklo Malaria Research Unit, Mahidol Oxford Tropical Medicine Research Unit, Mae Sot, Thailand; Shoklo Malaria Research Unit, Mahidol Oxford Tropical Medicine Research Unit, Mae Sot, Thailand; Shoklo Malaria Research Unit, Mahidol Oxford Tropical Medicine Research Unit, Mae Sot, Thailand; Shoklo Malaria Research Unit, Mahidol Oxford Tropical Medicine Research Unit, Mae Sot, Thailand; Shoklo Malaria Research Unit, Mahidol Oxford Tropical Medicine Research Unit, Mae Sot, Thailand; Centre for Tropical Medicine and Global Health, Nuffield Department of Medicine, University of Oxford, Oxford, United Kingdom; Mahidol Oxford Tropical Medicine Research Unit, Faculty of Tropical Medicine, Mahidol University, Bangkok, Thailand; Department of Population Health and Disease Prevention, University of California, Irvine, California, USA; Department of Epidemiology and Biostatistics, University of California, Irvine, California, USA; Shoklo Malaria Research Unit, Mahidol Oxford Tropical Medicine Research Unit, Mae Sot, Thailand; Faculty of Medicine, Institute of Global Health, University of Geneva, Geneva, Switzerland; Shoklo Malaria Research Unit, Mahidol Oxford Tropical Medicine Research Unit, Mae Sot, Thailand; Centre for Tropical Medicine and Global Health, Nuffield Department of Medicine, University of Oxford, Oxford, United Kingdom; Centre for Tropical Medicine and Global Health, Nuffield Department of Medicine, University of Oxford, Oxford, United Kingdom; Mahidol Oxford Tropical Medicine Research Unit, Faculty of Tropical Medicine, Mahidol University, Bangkok, Thailand

**Keywords:** epidemiology, malaria transmission, *Plasmodium vivax* relapse, primaquine, radical cure

## Abstract

**Background:**

In northwest Thailand, the provision of radical cure to prevent relapses of *Plasmodium vivax* malaria has decreased *P vivax* caseloads and decreased transmission. While malaria control measures were increased, we performed a prospective observational rolling cohort study to describe the changing incidence of *P vivax* malaria and the associated recurrence rates.

**Methods:**

Healthy nonpregnant glucose-6-phosphate dehydrogenase–normal volunteers who had symptomatic *P vivax* infection in the previous 12–24 months, but who had not received radical cure, were recruited. Supervised primaquine was given daily for 14 days (0.5 mg base/kg/day). Participants were followed 4 and 8 weeks later, then every 2 months until they developed symptomatic or asymptomatic *P vivax* malaria. Consultation for febrile illnesses was encouraged between follow-up visits. Participants who developed *P vivax* malaria were replaced with matched volunteers to maintain a continuous cohort of 200 participants.

**Results:**

From March 2010 until September 2014, 380 healthy adults and children were enrolled. Ninety-two individuals developed *P vivax* malaria, 25 within 4 months of enrollment. The annual incidence of *P vivax* malaria infection decreased from 0.19 in 2010 to 0.09 infections per person-year in 2014. The primaquine failure rate (*P vivax* malaria within 4 months of treatment) was 75% less than predicted based on earlier assessments that assumed a constant hypnozoite reservoir.

**Conclusions:**

Declining *P vivax* transmission reduces the hypnozoite reservoir in the population and the hypnozoite burden in an individual. This increases the apparent efficacy of radical cure in preelimination settings.

## Teaser

Without radical cure, young children bear the greatest burden of *Plasmodium vivax* relapse. Declining malaria transmission reduces the hypnozoite reservoir and thus the average number of relapses. Thus, apparent radical cure efficacy increases as *P vivax* prevalence declines.

*Plasmodium vivax* is now the main cause of malaria outside Africa. In much of Asia and the Americas, *P vivax* is increasingly targeted for elimination [1]. The mass distribution of insecticide-treated bed nets, insecticide spraying (in some areas), and, most importantly, the increasing availability of reliable and species-specific rapid diagnostic tests facilitating early diagnosis and treatment [2] have reduced the case incidence of *P vivax* malaria in Southeast Asia [3, 4], Oceania [5], and South America [6]. The majority of symptomatic *P vivax* malaria cases now result from relapses, which contribute to persistent malaria transmission. The blood schizonticides used to treat vivax malaria—chloroquine or artemisinin combination therapies—do not clear the hypnozoite stage, which causes relapses. The 8-aminoquinolines (primaquine or tafenoquine) are required to provide *P vivax* radical cure. But in patients with glucose-6-phosphate dehydrogenase (G6PD) deficiency, they cause iatrogenic hemolysis and may cause severe anemia [7]. Prior to the wider availability of the STANDARD G6PD Biosensor, G6PD testing was generally unavailable, so primaquine use has been inconsistent.

Frequent *P vivax* infections, which are common in parts of Southeast Asia and Oceania [8, 9], are associated with anemia [10], an increased risk of hospitalization [11], and death in young children [12]. *Plasmodium vivax* malaria in pregnancy results in small for gestational age newborns [13]. Maternal anemia caused by repeated *P vivax* malaria episodes increases the risk for stillbirth and neonatal death [14]. However, many *P vivax* infections are asymptomatic, reaching prevalences up to 50% in higher-transmission settings. Frequent relapses result in partial immunity [15–20] and persistent infection, contributing to ongoing transmission [21–23]. Preventing relapses is therefore necessary both for improving patient outcomes and interrupting transmission. Relapse cannot be distinguished reliably from a newly acquired infection, which makes determining the incidence of *P vivax* and force of infection in an endemic setting difficult. In 2010, to determine the incidence of *P vivax* malaria and describe epidemiologic changes after radical cure in a malaria-endemic region in northwest Thailand, we designed a prospective observational rolling cohort study to assess the incidence of *P vivax* malaria after giving primaquine to eliminate hypnozoites in previously infected healthy volunteers. This was conducted in parallel with chemotherapy studies to provide an estimate of the incidence of new infections in those studies, and thus the proportion of reinfections in recurrences.

## METHODS

### Patient Consent Statement

Written informed consent and assent (applicable for participants 7–18 years old) were obtained before conducting study activities. Ethics approval was given by the Mahidol Faculty of Tropical Medicine Ethics Committee (MUTM 2010-005, TMEC 09-082) in Thailand and the Oxford Tropical Research Ethics Committee (OXTREC 04-10) in the United Kingdom. This study was registered at ClinicalTrials.gov (NCT01076868).

### Study Setting

This study was conducted in an area of hill forest in northwest Thailand along the Myanmar border and included 5 field clinics that served migrant and refugee populations. The setting was similar to an epidemiologic study performed in the same region 20 years previously [24]. Although housing structures and lifestyle were largely unchanged, factors affecting the malaria ecology including implementation of malaria control measures, urban development, conflict, and population movement had changed (ca. 1991–2015) [25]. In general, malaria transmission intensities were heterogeneous between villages. Asymptomatic malaria prevalences (predominantly *P vivax*) ranged from 0 to 30% [26]. Malaria was the main cause of significant febrile illness. Increasing deployment of effective treatment and other control measures reduced malaria in the area [3]. Previously, G6PD testing was unavailable and primaquine was not prescribed routinely for *P vivax* malaria until 2017.

### Study Design

In this prospective observational study, we enrolled a rolling cohort of 200 healthy participants who had acute *P vivax* malaria documented in the previous 12 months and had not received primaquine radical cure. The cohort was recruited in categories defined by sex, age group, and recruitment site. When participants reached the study endpoint (symptomatic or asymptomatic *P vivax* malaria) they were replaced by sex-, age group–, and when possible, site-matched participants with a similar history of recent *P vivax* malaria. During the study period, declining *P vivax* caseloads reduced the pool of potential participants; thus, 15 months after the study started, the inclusion criterion for acute *P vivax* malaria in the previous 12 months was extended to 24 months.

A previous prospective epidemiologic study conducted in the same area from 1991 to 1992 [24] was used for historical comparison for *P vivax* recurrence rates. In that study, cross-sectional malaria surveys with blood smears were performed every 2–3 months in individuals of all ages within 127 randomly selected households. Recurrences following chloroquine-treated *P vivax* malaria were documented. Two vivax chemotherapy trials conducted in the area during 2010–2012 [8] and 2012–2015 [27] recruited from the same populations, had the same eligibility criteria and study duration, and had monthly follow-up. Malaria infections were diagnosed actively (routine follow-up) and passively (between visits). This observational study ran alongside the chemotherapy trials.

### Study Participants

Healthy nonpregnant volunteers with a history of blood smear–positive acute *P vivax* malaria were invited for screening if they were ≥6 months old and weighed ≥7 kg. Volunteers were excluded if they had G6PD deficiency, fever ≥37.5°C, hematocrit ≤25%, antimalarial treatment in the last 2 months, or current microscopy-detected malaria infection, or if they were breastfeeding an infant <6 months old.

### Study Procedures

The following were performed: complete medical history and physical examination, blood film, hematocrit, complete blood count, G6PD fluorescent spot test, and urine pregnancy test if applicable. Enrolled participants received a supervised weight-based primaquine dose (0.5 mg base/kg administered after food) (Government Pharmaceutical Organization, Thailand) once daily for 14 days. A full dose was repeated if vomiting occurred within 30 minutes and a half dose was repeated if within 1 hour. Adverse events were recorded during the study drug treatment.

### Follow-up

After enrollment, participants were followed at weeks 4 and 8, then every 2 months thereafter. At each visit a medical history, physical examination, malaria blood film, capillary hematocrit, and urine pregnancy test (if applicable) were performed. Participants were encouraged to return in between follow-up visits for febrile illnesses. As this study was conducted over 5 years, participants planning to leave the area were encouraged to notify the study team so a final visit could be performed.

### Sample Size

In 2009, the incidence of *P vivax* malaria was an estimated 0.20 per person-year (ppy). Thus, 200 healthy volunteers would enable the measurement of a 20% incidence per year with ±6% precision and 95% confidence. To maintain the estimated incidence precision in the cohort, participants who developed malaria or left the study were replaced with a matched volunteer.

## Statistical Analysis

The primary endpoint of this study was the incidence of *P vivax* malaria (symptomatic or asymptomatic) after radical cure. However, relapse cannot be reliably distinguished from new infection in endemic areas. As treatment efficacy studies in this same population have shown that nearly all relapses occur within 4 months [8], we considered all *P vivax* recurrences before 4 months to be primaquine failures. Modeling from detailed malaria surveys conducted in the same area estimated the mean duration of carriage for a hypnozoite to be 6 months [28].

Overall incidence rates were analyzed using Kaplan-Meier analysis and Poisson regression, then repeated designating those leaving the study <4 months as having *P vivax* malaria. The overall incidence of *P vivax* malaria was compared to the historical incidence (described above). The time to *P vivax* recurrence after radical cure stratified by vivax malaria history at <12 months versus ≥12 months was assessed using Kaplan-Meier method with Cox regression. The proportional hazards assumption was tested using the *estat phtest* command in StataNow. As relapse risk would be lower for participants with a more distant vivax history, a sensitivity analysis was performed to determine whether there was a difference between a vivax malaria history at <12 months versus ≥12 months for *P vivax* recurrence at both 4- and 6-month timepoints. A sensitivity analysis was also performed to determine whether there was a difference between *P vivax* recurrence at 4 versus 6 months.

Multivariable logistic regression was used to determine risk factors for primaquine failure (*P vivax* <4 months) and reinfection (*P vivax* ≥4 months), including the recurrence year, age group, sex, migrant or refugee status, distance living away from the clinic, occupation, body mass index, and hematocrit. Distance in kilometers between the patients’ home village and recruitment clinic was estimated using geographical coordinates (QGIS version 3.28). Hematocrit differences were assessed using multivariable linear regression with body mass index and migrant or refugee status as covariates. Follow-up and adverse events were described.

We previously reported a probabilistic model from the concomitant chemotherapy trials (described above), which used genotyping and time to recurrence to distinguish relapse from new infection and estimate the vivax relapse rate [29]. An estimated incidence rate ratio (IRR) was calculated using the *P vivax* malaria relapse rates following chloroquine treatment with versus without primaquine radical cure. A comparison was made post hoc between the primaquine failure rate from this study and from the probabilistic model. Data analysis was performed using Stata 18.0 and StataNow 19.5.

## RESULTS

### Screening and Enrollment of the Initial Cohort and Replacements

Over 6 weeks from March to April 2010, 200 participants were recruited (Supplementary Figure 1). The most common reason for exclusion during screening was a positive malaria smear; 213 of 880 (24%) were positive for *P vivax* and 8 (1%) for *Plasmodium falciparum*. Overall, between March 2010 and September 2014, 380 participants were recruited and 180 were replaced after meeting the study endpoint (ie, developed *P vivax* recurrence).

### Participant Characteristics

Participant characteristics were similar between the excluded and followed participants (Table 1 and Supplementary Table 1). The demographics of the enrolled volunteers were similar to the overall outpatient malaria data from the same years (Supplementary Tables 2 and 3). Most adults were farmers (152/203 [75%]). Approximately half of the children 5–15 years old attended school (62/127 [49%]). Baseline hematocrit was lower in females compared to males (mean, 2.8% lower [95% confidence interval {CI}, 2.1%–3.5%]; *P* < .001), and children had a lower baseline hematocrit than adults: age group 0–4 years (mean, 4.7% lower [95% CI, 3.4%–6.0%; *P* < .001) and 5–15 years (mean, 2.0% lower [95% CI, 1.0%–3.1%]; *P* < .001). The spleen index was less than 1%. The overall time from the previous vivax malaria episode to enrollment was 225 days (interquartile range [IQR], 130–287 days; range, 2–694 days) (Supplementary Table 4 and Supplementary Figure 2).

**Table 1.** Participant Characteristics

Participant Characteristics	Age Group		
	0–4 y (n = 50)	5–15 y (n = 127)	>15 y (n = 203)
Female sex	25 (50)	64 (50)	78 (38)
Site			
Maw Ker Thai	14 (28)	40 (31)	47 (23)
Maela	1 (2)	10 (8)	21 (10)
Murunchai	4 (8)	6 (5)	5 (3)
Mae Khon Khen	3 (6)	17 (13)	29 (14)
Wangpha	28 (56)	54 (43)	101 (50)
Occupation			
Farming	0	25 (20)	152 (75)
Forest worker	0	3 (2)	7 (3)
Work at home	0	11 (9)	21 (10)
Attending school	4 (8)	62 (49)	5 (3)
Factory	0	2 (1)	5 (3)
Follow parent to work	5 (10)	4 (3)	0
Other	0	0	3 (1)
Not working	41 (82)	20 (16)	10 (5)
Ethnicity			
Burman	7 (14)	16 (12)	63 (31)
Karen	41 (82)	100 (79)	132 (65)
Mixed	2 (4)	6 (5)	4 (2)
Other	0	5 (4)	4 (2)
Distance^a^ from clinic, km, median (range)	4.7 (0.5–22.7)	2.1 (0.5–10.6)	1.8 (0.5–4.2)
Weight, kg, mean (range)	11.6 (7.5–21)	27.4 (10–55)	50.4 (21–72)
BMI, kg/m^2^, mean (range)	16 (12.6–26.5)	16 (11.1–23.8)	20 (14.6–36.8) (n = 2 missing)
Temperature, ℃, mean (range)	37.0 (36.0–37.5)	36.8 (36.0–37.5)	36.8 (36–37.4)
Heart rate, minimum, mean (range)	105 (74–132)	88 (62–133)	76 (52–100)
Respiratory rate, minimum, mean (range)	32 (20–48)	25 (18–36)	23 (16–32)
Splenomegaly	0	0	1 (1)
Hepatomegaly	2 (4)	2 (2)	2 (1)
Day 0 hematocrit (%) overall, mean (range)	35 (30–41)	38 (31–48)	41 (31–52)
Day 0 hematocrit (%) in males, mean (range)	35 (30–41)	39 (31–48)	43 (31–51)
Day 0 hematocrit (%) in females^b^, mean (range)	36 (31–40)	37 (31–43)	38 (31–52)
Absolute hematocrit (%) change^c^ in males, mean (range)	−0.04 (−9 to 12)	−1 (−12 to 20) (n = 1 missing)	−1 (−10 to 14) (n = 1 missing)
Absolute hematocrit (%) change in females^b^, mean (range)	−1 (−6 to 5)	−3 (−12 to 5) (n = 1 missing)	−2 (−12 to 7) (n = 1 missing)
Time from most recent Pv malaria before enrollment, d, median (IQR, range)	175 (119–278, 65–633)	238 (139–295, 13–655)	222 (139–285, 2–694)
Pv malaria recurrence <4 mo after enrollment	6 (12)	7 (6)	12 (6)
Follow-up duration, d, median (IQR, range)	853 (392–1571, 47–1618)	448 (225–953, 27–1625)	561 (224–1067, 26–1631)
Left the study <4 mo from enrollment	0	9 (7)	20 (10)
Males leaving the study <4 mo^d^	0	5 (56)	13 (65)

Data are presented as No. (%) unless otherwise indicated.

Abbreviations: BMI, body mass index; IQR, interquartile range; d, day; Pv, *Plasmodium vivax*.

^a^Distance between the patients’ home village and recruitment clinic.

^b^Hematocrit analysis in females includes an additional 2 glucose-6-phosphate dehydrogenase heterozygous Mahidol (phenotype normal) adult females who completed 14 days of primaquine but were excluded from the remaining analyses.

^c^Hematocrit change is the absolute difference between hematocrit values on days 0 and 14.

^d^Percentage is calculated from the total participants lost to follow-up.

### Annual Incidence of *P vivax* Recurrence

The overall incidence of *P vivax* malaria recurrence following primaquine radical cure (ie, relapses and reinfections) was 0.13 ppy (Table 2). The incidence decreased each year by approximately 31% from 2010 to 2014 (IRR, 0.69 [95% CI, .60–.78]; *P* < .001). In 2010, children 0–4 years old had the overall highest incidence of *P vivax* malaria at 0.29 ppy (Supplementary Table 5). By 2014 this halved to 0.14 ppy. This reduction was not significantly different from that in older children (IRR, 0.8 [95% CI, .4–1.3]; *P* = .3) or adults (hazard ratio, 0.7 [95% CI, .4–.9]; *P* = .1). Females had a lower annual incidence of *P vivax* malaria than males (IRR, 0.6 [95% CI, .4–.9]; *P* = .02). When designating participants leaving the study within 4 months as primaquine failure, the results were similar. Compared to the study conducted in 1991–1992 [24], the incidence of *P vivax* in this study (20 years later) in children 0–4 years was over 6 times lower and in the older age groups was 1–2 times lower than previously observed (Supplementary Figures 3 and 4). The annual incidence of *P vivax* during the dry seasons decreased more rapidly (IRR, 0.4 [95% CI, .2–.6]; *P* < .0001) than during the rainy seasons (Supplementary Table 6).

**Table 2.** Annual Incidence of *Plasmodium vivax* Malaria in Healthy Volunteers After Daily High-Dose Primaquine Monotherapy (7 mg Base/kg Total Dose) Radical Cure From March 2010 to September 2014

Year	Participants Enrolled, No.	Participants Followed, No.	Pv Cases	Follow-up Time (ppy)	Overall Incidence (ppy)	(95% CI)	Incidence Within 4 Months (ppy)
2010	231	231	28	146.1	0.19	(.13–.28)	0.14
2011	53	219	20	165.7	0.12	(.08–.19)	0.02
2012	57	223	16	158.6	0.10	(.06–.16)	0.01
2013	32	207	21	153.8	0.13	(.09–.21)	0
2014	7	159	7	79.2	0.09	(.04–.18)	0
Overall	380	380	92	703.4	0.13	(.11–.16)	0.04

Abbreviations: CI, confidence interval; ppy, per person-year; Pv, *Plasmodium vivax*.

### Time to *P vivax* Recurrence

The median interval to *P vivax* recurrence after enrollment for participants with a vivax history <12 months was 335 days (IQR, 112–588 days; range, 27–1557 days) and when ≥12 months was 301 days (IQR, 224–605 days; range, 130–630 days). When comparing the recurrence-free probability between these 2 groups (Supplementary Figure 5), persons with a vivax history ≥12 months had a higher probability of being recurrence free at 10 months of follow-up. After this time, the recurrence-free probabilities in both groups were similar irrespective of the timing of vivax history. The proportional hazard assumption test held (χ^2^ = 0.00, degrees of freedom = 1, *P* = 1.0). Sensitivity analysis showed no difference in the probability of primaquine failure (recurrence <4 months from enrollment) when comparing participants with a vivax history <12 months or ≥12 months before enrollment (*z*-statistic −0.62; *P* = .5).

### Primaquine Failure (*P vivax* Recurrence Within 4 Months)

**Figure 1.**
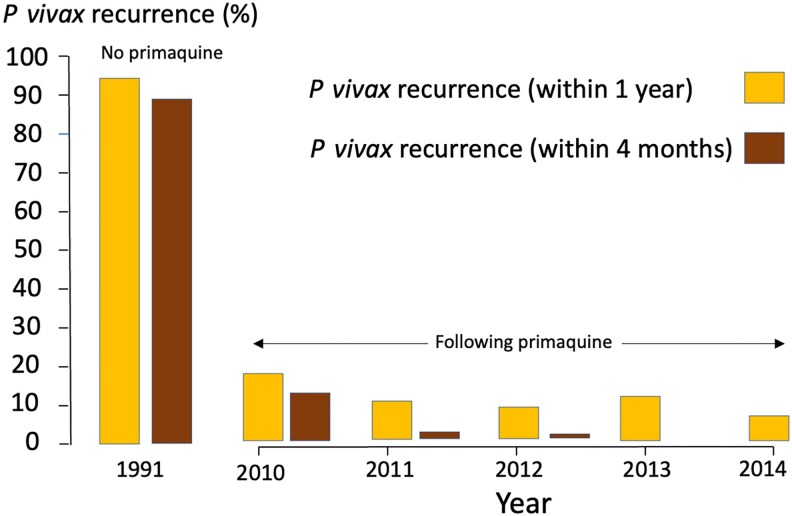
Comparison of contemporary (after primaquine) and historical (without primaquine) incidence of *Plasmodium vivax* malaria.

At 4 months, the incidence of *P vivax* malaria following primaquine monotherapy was 0.04 ppy. The annual primaquine failure rate decreased over the years 2010, 2011, and 2012 (0.14, 0.02, and 0.01 ppy, respectively; Table 2 and Figure 1) to a greater extent than recurrences occurring within 1 year. There was an approximately 83% reduction in primaquine failure from 2010 to 2014 (IRR, 0.17 [95% CI, .08–.39]; *P* < .001). Sensitivity analysis showed no difference between the probability of primaquine failure if recurrences were assessed at 4 or 6 months (*z*-statistic −0.46; *P* = .6).

### Factors Associated With *P vivax* Malaria Recurrences

**Figure 2.**
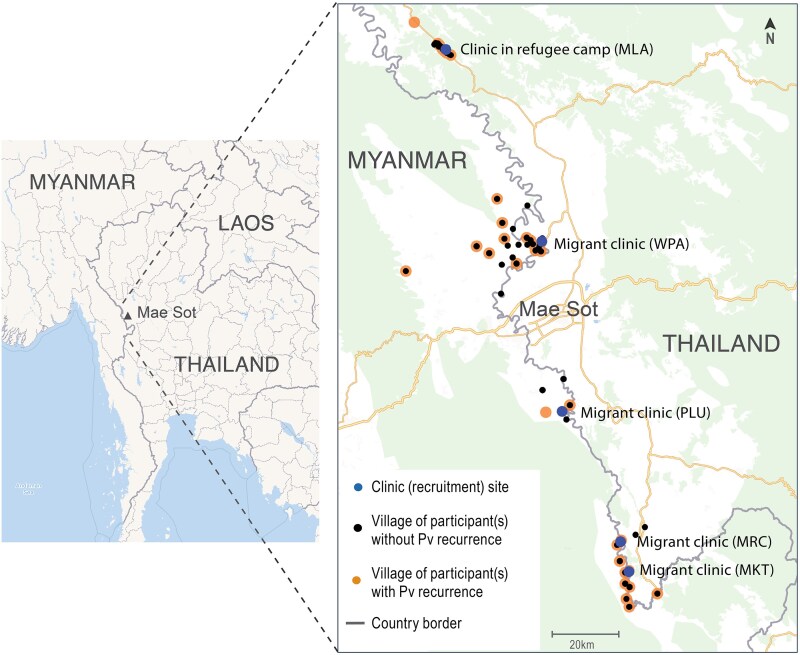
Map of participants’ villages showing locations of those with and without *Plasmodium vivax* recurrence. Abbreviations: MKT, Maw Ker Thai; MLA, Maela; MRC, Murunchai; PLU, Mae Khon Khen; Pv, *Plasmodium vivax*; WPA, Wangpha. Adapted by the authors using map data from OpenStreetMap www.openstreetmap.org under the Open Database Licence https://opendatacommons.org/licenses/odbl/1-0/, and from the database ‘Villages and GPS points.xlsx’ from the Shoklo Malaria Research Unit. All rights reserved. Permission for re-use should be sought from the rights-holder.

The only risk factor for *P vivax* malaria at <4 months (ie, primaquine failure) was being male (odds ratio [OR], 3.6 [95% CI, 1.2–10.5]; *P* = .02) (Supplementary Table 7). Participants with *P vivax* malaria ≥4 months (ie, reinfections) were more likely to live in villages >10 km from the clinic (OR, 2.7 [95% CI, 1.1–7.0]; *P* = .03) than those who did not (Figure 2). Being male was not a risk factor for *P vivax* malaria ≥4 months (Supplementary Table 8). Age, migrant or refugee status, occupation, body mass index, and enrollment hematocrit were not risk factors for either (Supplementary Tables 7 and 8). As the study progressed, the cohort replacements came from villages increasingly farther away (mean, 2.8 km [95% CI, .7–4.9]; *P* = .01) than the initial enrollee. Village locations were similar in the parallel chemotherapy trials (Supplementary Figure 6).

### Follow-up

Overall, the median follow-up duration was 561 days (IQR, 225–1122 days; range, 26–1631 days) with most participants being followed for >1 year (Table 1) and according to scheduled visits (median, 45 days between visits [IQR, 37–56 days; range, 13–634 days]) (Supplementary Table 9*A*). Of participants leaving the study early, 76 of 178 (43%) left before completing their first year (Supplementary Table 9*B* and 9*C*). Of the remaining 102 of 178 participants, the majority (77/102 [75%]) adhered to scheduled visits in their first year (median, 42 days between visits [IQR, 37–42 days; range, 28–62 days) but left during their second follow-up year (Supplementary Table 9*D*). All participants completed study activities on their last follow-up day. There were no differences in follow-up duration between the study sites.

### Adverse Events

The mean hematocrit at enrollment was lower in females and in children 0–4 years old (Table 1). This difference persisted at day 14 only for females (mean hematocrit, 6.1% lower than males [95% CI, 3.9%–8.5%]; *P* < .001). Of 150 adverse events during primaquine treatment (in 111 participants), there were 8 (5%) complaints of epigastric pain, 2 (1%) of anorexia or vomiting, and 9 (6%) of headache or dizziness. Anemia (hematocrit <30%) was treated in 5 (3%) participants, of whom 3 were male. Two participants stopped primaquine because of elevated methemoglobin levels. They recovered uneventfully. The remaining adverse events were from mild infections or alternative diagnoses. Three deaths (suicide, homicide, and an unknown cause) unrelated to the study drug were reported, occurring 6 months to 2 years after enrollment.

### Post Hoc Comparison of Primaquine Failure Rates

We previously estimated the reinfection-adjusted primaquine failure rate to be 0.04 ppy following chloroquine and primaquine, and that following chloroquine monotherapy to be 3.3 ppy—an IRR of 0.012 (ie, IRR = 0.04/patient-year ÷ 3.3/patient-year). When compared to the probability model, by 2012, the primaquine failure in this study was approximately 75% less than predicted (Table 3).

**Table 3.** Comparison of the Incidence Rate Ratios for *Plasmodium vivax* Relapse in the Rolling Cohort Study and Probabilistic Model

Probability Model	Reinfection-Adjusted Failure Rate (ppy) After CQ + PQ	Reinfection-Adjusted Relapse Rate (ppy) After CQ Monotherapy^a^	Incidence Rate Ratio
Taylor et al, 2019 [29]	0.04	3.3	0.012
Year of follow-up in the rolling cohort study	Primaquine failure rate (ppy) after PQ monotherapy^b^		
2010	0.14	3.3	0.042
2011	0.02	3.3	0.006
2012	0.01	3.3	0.003

Abbreviations: CQ, chloroquine; ppy, per person-year; PQ, primaquine.

^a^Original data are from Nosten et al [13] and White [32] (the parallel vivax chemotherapy trials).

^b^ *Plasmodium vivax* recurrences within 4 months of treatment in the rolling cohort were considered primaquine failure.

## DISCUSSION

Prospective malaria studies conducted in the population living along the Thailand–Myanmar border over the past 30 years have enhanced substantially our understanding of *P vivax* epidemiology and biology [8, 26–28, 30]. In Southeast Asia, *P vivax* infections relapse frequently. In the 1990s, the recorded case incidence of *P vivax* was high in this area, as it probably had been throughout history. Twenty years later, as continued malaria control activities (mainly provision of rapid diagnosis and early effective antimalarial treatment) reduced malaria, the case incidence of *P vivax* malaria fell. However, relapse rates reflect an individual's previously acquired hypnozoite burden so, without radical cure, recurrence rates did not fall concomitantly. Because of the hysteresis, as the incidence of *P vivax* declined, the relative contribution of repeated relapses to overall recurrence increased [31]. There is evidence that *P falciparum* malaria activates hypnozoites, so concomitant elimination of *P falciparum* may also have contributed to a decrease in *P vivax* [32–34] recurrence. As the population hypnozoite reservoir was depleted, the incidence of infection fell further.

In endemic areas, children 0–4 years old have a much greater risk of relapse than older children and adults [24], so they gain the greatest benefit from effective radical cure. In this study, supervised radical cure prevented *P vivax* recurrence across all age groups and ethnicities, irrespective of migratory or refugee status and body mass index (a proxy for malnutrition and poverty). However, men continued to have higher risks for *P vivax* <4 months after radical cure (primaquine failure), possibly because they had higher individual hypnozoite loads from previous high exposure to malaria in their work environments. Men had higher risks for *P vivax* malaria <4 months, but afterward their risks were not increased, possibly because the males remaining in follow-up lived and worked closer to the clinic where malaria transmission was declining. Replacing males in the cohort was challenging as those with a vivax malaria history came from farther away and often could not participate in the study.

The recurrence rate <4 months following radical cure in this study (defined as primaquine failure, but in other studies defined as relapse) was much lower than recorded previously, and 75% lower than the predicted primaquine failure rate in a probability model [29] developed from contemporaneous data that assume a constant population hypnozoite burden over time. The relapse risk reflects the intensity of exposure largely over the previous year [28]. The observed difference therefore presumably reflects the continued population reduction in hypnozoite loads [35, 36]. The relationship between the population's hypnozoite reservoir and the radical curative efficacy of primaquine is nonlinear. This is because only 1 activated hypnozoite is required to produce a relapse [37]. The distribution of hypnozoites in the population is uneven (overdispersed) [28, 36]. Within an individual, the radical cure efficacy required to remove all hypnozoites declines as their numbers decrease.

There are several limitations to this study. Over time, *P vivax* malaria rates in the study population reflected rapid changes in malaria epidemiology. Participants may have had submicroscopic malaria at enrollment and during the study, which was not detected, thus underestimating *P vivax* infections. The initial radical cure primaquine regimen, with its weak schizonticidal activity, may not have eliminated those infections. However, in this study, only 1 participant developed *P vivax* infection within 14 days of completing primaquine at day 27. If the last vivax malaria episode was ≥12 months previous, this may indicate a low risk of infection and thus a lower hypnozoite burden, which would decrease primaquine failure rates. On the other hand, assuming that all recurrences <4 months are relapses would overestimate primaquine failure rates as some of the recurrences could be new infections.

In this study, other factors may have contributed to a lower measured incidence of *P vivax* malaria. More females remained in study follow-up. It was difficult replacing healthy males in the study due to their work obligations. This may have caused a falsely low reinfection rate in that group as lower-risk males remained in follow-up. Patients with acute *P vivax* malaria came from increasingly distant villages, so over time the cohort was skewed toward those who could be followed up (ie, those living or working closer to the recruitment clinics).

Declining *P vivax* transmission reduces the population hypnozoite reservoir, thus increasing the apparent efficacy of radical cure in high-risk populations. As an individual's hypnozoite burden decreases, so does the risk of relapse after radical cure. Higher doses of 8-aminoquinolines may not be needed in preelimination settings.

## Supplementary Material

ofaf667_Supplementary_Data

## Supplementary Data

Supplementary materials are available at *Open Forum Infectious Diseases* online. Consisting of data provided by the authors to benefit the reader, the posted materials are not copyedited and are the sole responsibility of the authors, so questions or comments should be addressed to the corresponding author.
