# Supplementary material for: Plasmodium vivax Malaria Relapse Risk Depends on Transmission Intensity: Evidence From a Longitudinal Study in Northwest Thailand
Source: Open Forum Infect Dis. 2025 Oct 30;13(1):ofaf667. doi: 10.1093/ofid/ofaf667 (PMC12803024; doi:10.1093/ofid/ofaf667)
Supplement: ofaf667_Supplementary_Data [file ofaf667_supplementary_data.pdf]

SUPPLEMENT FILES

Supplement Table 1. Participant characteristics in those excluded after enrollment

Participant characteristics	Participants excluded after enrollment		
	Age group		
	0-4 years	5-15 years	>15 years
	n=3	n=4	n=15
Male; n (%)	1 (33)	3 (75)	10 (67)
Site; n (%)			
Maw Ker Thai	2 (67)	1 (25)	5 (33)
Maela	0	1 (25)	1 (7)
Morunchai	0	0	1 (7)
Mae Khon Khen	0	2 (50)	3 (20)
Wangpha	1 (33)	0	5 (33)
Occupation; n (%)			
Farming	0	2 (50)	12 (80)
Forest	0	0	2 (13)
Work at home	1 (33)	0	0
Attend school	0	3 (75)	0
Factory	0	0	0
Follow parent to work (small children)	0	0	0
Other	0	0	0
Not working	2 (67)	1 (25)	1 (7)
Ethnicity; n (%)			
Burmese	0	0	8 (53)
Karen	3 (100)	3 (75)	7 (47)
Mixed	0	1 (25)	0
Other	0	0	0
Weight; median kg (range)	12 (9-15)	31 (21-55)	50 (40-59)
BMI; median (range)	17.8 (14.4-23.4)	15.6 (13.7-20.2)	19.8 (16.4-21.9)
Temperature; median ℃ (range)	37.2 (36.7-37.2)	36.6 (36.2-37.4)	36.8 (36.0-37.4)
Heart rate; median bpm (range)	104 (98-112)	78 (70-86)	80 (60-96)
Respiratory rate; median rpm (range)	28 (26-52)	23 (22-28)	22 (18-26)
Splenomegaly; n (%)	0	0	0
Hepatomegaly; n (%)	0	0	1 (7)
Day 0 haematocrit (%), median (range)	39 (33-42)	37 (31-48)	43 (32-48)
Day 0 haematocrit (%) in males, median (range)	39* (NA)	37 (37-48)	46 (32-48)
Day 0 haematocrit (%) in females; median	38 (33-42)	31* (NA)	41 (37-42)

* There is only one participant in this group

Supplement Table 2. Description of the proposed cohort, the participants who completed the study, and outpatient consultations for Plasmodium vivax malaria during the study period, stratified by age and sex

	Proposed cohort of 200 based on outpatient consultations from 2008-09[a]			Actual completed cohort of 380 in this study			Outpatient consultations for P. vivax malaria from 2010-2014[b]		
Age group	0-4 year	5-15 year	>15 year	0-4 year	5-15 year	>15 year	0-4 year	5-15 year	>15 year
	n=38	n=52	n=110	n=50	n=127	n=203	n=1,720	n=3,804	n=6,669
Male; n(%)	20 (53)	29 (56)	69 (63)	25 (50)	63 (50)	125 (62)	864 (50)	2,291 (60)	4,741 (71)
Site; n(%)									
MKT	8 (21)	11 (21)	15 (14)	14 (28)	40 (31)	47 (23)	485 (28)	1,230 (32)	1,952 (29)
MLA	1 (2)	5 (10)	9 (8)	1 (2)	10 (8)	21 (10)	27 (2)	148 (4)	261 (4)
MRC	4 (11)	5 (10)	6 (5)	4 (8)	6 (5)	5 (2)	242 (14)	627 (16)	628 (9)
PLU	3 (8)	5 (10)	14 (13)	3 (6)	17 (13)	29 (14)	107 (6)	486 (13)	1,174 (18)
WPA	22 (58)	26 (50)	66 (60)	28 (56)	54 (43)	101 (50)	859 (50)	1,313 (35)	2,654 (40)

[a]The cohort was pre-defined by sex, age group, and recruitment site to correspond to the population presenting to the clinics for consultation in 2008-2009
[b]The epidemiology of *P. vivax* malaria changed during this time so outpatient vivax consultations from 2010-2014 (same years as the study) are presented for comparison

Supplement Table 3. Clinic site distribution of the proposed cohort, the participants who completed the study, and outpatient consultations for *Plasmodium vivax* malaria during the study period

Clinic site; n(%)	Proposed cohort of 200 based on outpatient consultations from 2008-09	Completed cohort of 380 in this study	Outpatient consultations for *P. vivax* malaria from 2010-2014*
MKT	34 (17)	101 (27)	3,667 (30)
MLA	15 (8)	32 (8)	436 (4)
MRC	15 (8)	15 (4)	1,497 (12)
PLU	22 (11)	49 (13)	1,767 (14)
WPA	114 (57)	183 (48)	4,826 (40)
Total	200 (100)	380 (100)	12,193 (100)

*The epidemiology of *P. vivax* malaria changed during this time so outpatient vivax consultations from 2010-2014 (same years as the study) are presented for comparison

Supplement Table 4. The most recent documented history of *Plasmodium vivax* malaria infection prior to enrollment in the study

Most recent P. vivax infection prior to enrollment	Number of participants (% of total)
0 to <4 months	89 (23)
4 to <6 months	64 (17)
6 to <9 months	110 (29)
9 to <12 months	88 (23)
12 to <24 months	29 (8)
Total	380 (100)

Supplement Table 5. Annual incidence of *Plasmodium vivax* malaria (symptomatic and asymptomatic) from March 2010 to September 2014 by age group

Years	Participants 0-4 years old in follow up, n (%)	*P. vivax* cases	Follow up time (person-year)	Incidence (person-year)	95% CI
2010	37 (16)	7	24.3	0.29	0.14-0.60
2011	34 (16)	2	28.6	0.07	0.02-0.28
2012	38 (17)	2	29.6	0.07	0.02-0.27
2013	38 (18)	5	31.8	0.16	0.06-0.38
2014	28 (18)	2	14.2	0.14	0.04-0.56
Overall	52 (14)	18	128.4	0.14	0.09-0.22

Years	Participants 5-15 years old in follow up, n (%)	*P. vivax* cases	Follow up time (person-year)	Incidence (person-year)	95% CI
2010	69 (30)	6	41.7	0.14	0.06-0.32
2011	64 (29)	8	47.6	0.17	0.08-0.34
2012	66 (30)	7	45.9	0.15	0.07-0.32
2013	69 (33)	7	48.8	0.14	0.07-0.30
2014	58 (36)	3	29.7	0.10	0.03-0.31
Overall	125 (33)	31	213.8	0.12	0.08-0.16

Years	Participants >15 years old in follow up, n (%)	*P. vivax* cases	Follow up time (person-year)	Incidence (person-year)	95% CI
2010	125 (54)	15	80.0	0.19	0.11-0.31
2011	121 (55)	10	89.4	0.11	0.06-0.21
2012	119 (53)	7	83.1	0.08	0.04-0.18
2013	100 (48)	9	73.2	0.12	0.06-0.24
2014	73 (46)	2	35.3	0.06	0.01-0.23
Overall	203 (53)	43	361.1	0.13	0.10-0.16

Supplement Table 6. Annual incidence of *Plasmodium vivax* malaria (actively and passively detected) from March 2010 to September 2014 during the rainy season and the following dry season

Rainy season	Follow up time in person-years	*P. vivax* cases in the rainy season[a]	Incidence per person-years	95% CI	Dry Season	Follow up time in person-years	*P. vivax* cases in the following dry season[a]	Incidence per person-years	95% CI	IRR[b] between dry and rainy seasons
Apr-Aug 2010	78.4	21	0.27	0.17-0.41	Sep10-Mar11	98.9	10	0.10	0.05-0.19	0.37
Apr-Aug 2011	68.0	14	0.21	0.12-0.35	Sep11-Mar12	96.2	6	0.06	0.03-0.14	0.29
Apr-Aug 2012	62.0	12	0.19	0.11-0.34	Sep12-Mar13	97.5	1	0.01	0.001-0.07	0.05
Apr-Aug 2013	62.3	20	0.32	0.20-0.49	Sep13-Mar14	84.1	2	0.02	0.006-0.10	0.07
Apr-Aug 2014[c]	42.9	6	0.14	0.06-0.31	-	NA	NA	NA	-	0.00

[a]The rainy season was 5 months (April to August) and the following dry season was 7 months (September to March the following year).
[b]IRR (Incidence rate ratio) was calculated between the incidence of the dry season to the previous rainy season. For example, Sep10-Mar11 versus Apr-Aug 2010 is 0.10/0.27=0.37.
[c]The study follow up was stopped in July 2014 and the last follow up of the last participant was in September 2014.

Supplement Table 7. Risk factors for *Plasmodium vivax* recurrence <4 months (primaquine failure) after enrollment

	Odds Ratio	95% CI	p-value
Age group			
0 to 4 years	0.53	0.05 to 5.2	0.6
5 to 15 years	0.62	0.12 to 3.1	0.6
>15 years	comparator		
Sex			
Male	3.58	1.2 to 10.5	0.02
Female	comparator		
Living >10km from the clinic	0.64	0.13 to 3.2	0.59
Migrant status (refugee)			
Refugee	0.59	0.05 to 7.2	0.7
Migrant	comparator		
Occupation			
Farmer	0.67	0.10 to 4.5	0.7
Woodcutter	0.32	0.02 to 5.1	0.4
Stay at home	0.44	0.03 to 6.0	0.5
Student	1.25	0.20 to 7.9	0.8
Follow parents to work (small children)	1.29	0.12 to 14.2	0.8
Body Mass Index	0.91	0.78 to 1.1	0.2
Baseline hematocrit	1.02	0.90 to 1.2	0.7

Supplement Table 8. Risk factors for *Plasmodium vivax* recurrence ≥4 months (re-infection) after enrollment

	Odds Ratio	95% CI	p-value
Age group			
0 to 4 years	1.6	0.38 to 6.9	0.5
5 to 15 years	1.6	0.67 to 4.0	0.3
>15 years	comparator		
Sex			
Male	1.6	0.87 to 3.1	0.1
Female	comparator		
Living >10km from the clinic	2.7	1.1 to 7.0	0.03
Migrant status (refugee)			
Refugee	0.8	0.23 to 2.7	0.7
Migrant	comparator		
Occupation			
Farmer	1.1	0.33 to 3.7	0.9
Woodcutter	1.2	0.23 to 6.5	0.8
Stay at home	0.9	0.18 to 4.5	0.9
Student	0.9	0.28 to 2.7	0.8
Other	15.7	0.57 to 433	0.1
Body Mass Index	1	0.94 to 1.1	0.8
Baseline hematocrit	0.9	0.86 to 1.0	0.1

Supplement Table 9a. Follow up frequency during each year of the study after enrollment

	Year 1	Year 2	Year 3	Year 4	Year 5	Total
Median days between follow up (IQR, range)	37 (31-42, 13-74)	55 (48-57, 25-171)	56 (49-60, 32-279)	56 (49-65, 28-383)	56 (46-64, 28-269)	45 (37-56, 13-383)

The follow up year begins from enrollment. In Year 1 there are more frequent follow up visits (weeks 2, 4 and 8, then every 2 months).

Supplement Table 9b. Participants leaving the study overall, stratified by age group and sex

Sex	Age group			Total
	0-4 years	5-15 years	>15 years	
Male (%)	5 (42)	18 (39)	72 (60)	95 (53)
Female (%)	7 (58)	28 (61)	48 (40)	83 (47)
Total (%)	12 (100)	46 (100)	120 (100)	178 (100)

Supplement Table 9c. Participants leaving the study before one year, stratified by age group and sex

Sex	Age group			Total
	0-4 years	5-15 years	>15 years	
Male (%)	0	10 (50)	38 (68)	48 (63)
Female (%)	0	10 (50)	18 (32)	28 (37)
Total (%)	0	20 (100)	56 (100)	76 (100)

Supplement Table 9d. Follow up frequency in participants leaving the study before and after one year

	PD	Year 1	Year 2	Year 3	Year 4	Year 5	Total
		n=76					n=76
Follow up frequency; median days (IQR, range)	Before 1 year	31 (23-38, 13-74)	NA	NA	NA	NA	31 (23-38, 13-74)
		n=77	n=8	n=6	n=7	n=4	n=102
	After 1 year	42 (37-42, 28-62)	57 (56-71, 42-171)	208 (75-253, 60-279)	56 (51-136, 42-383)	139 (80-208, 28-269)	42 (37-48, 28-383)

Premature discontinuation (PD)

Supplement Figure 1. Trial diagram

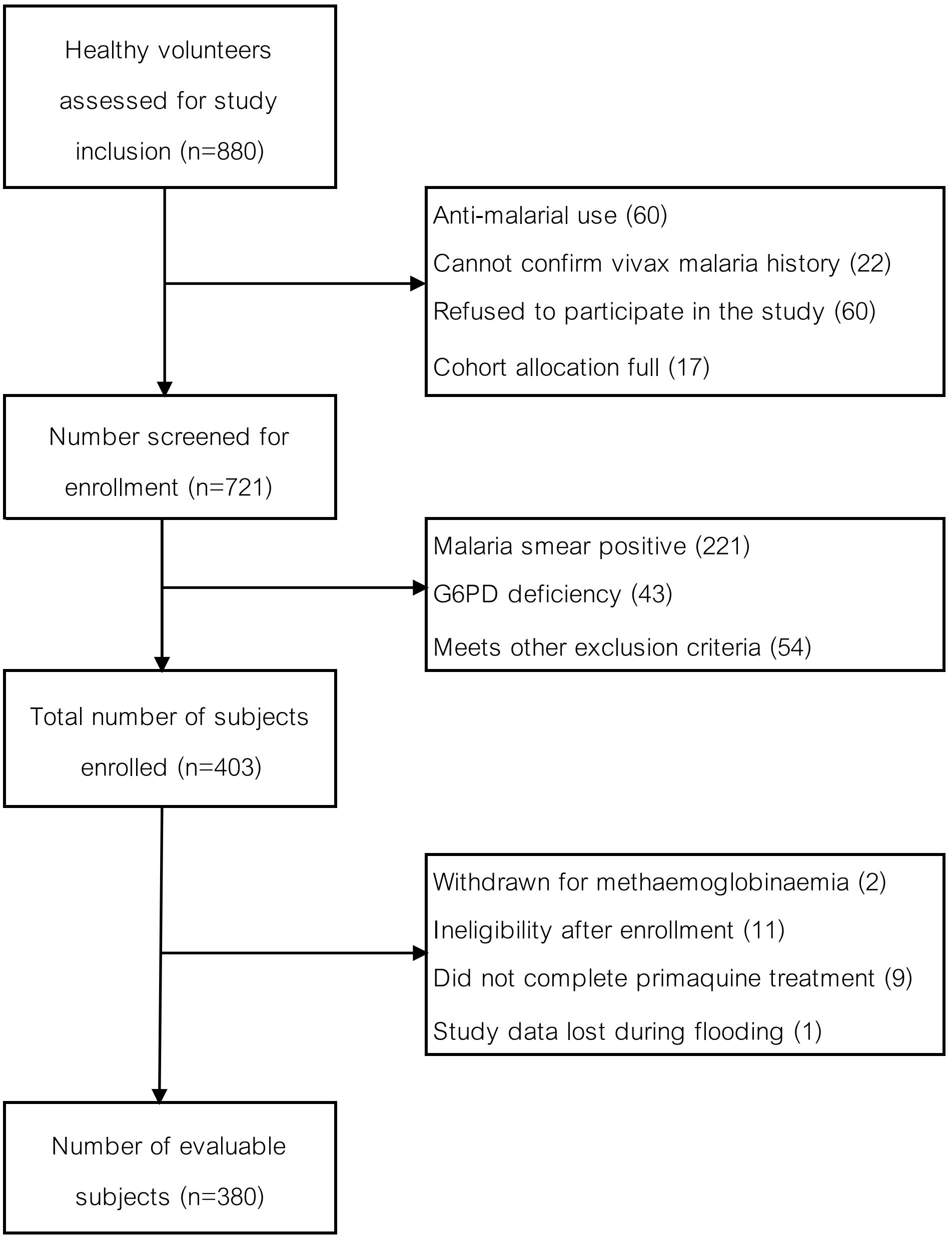

Supplement Figure 2. Median number of months between the most recent documented history of *Plasmodium vivax* malaria infection and study enrollment stratified by age group and sex

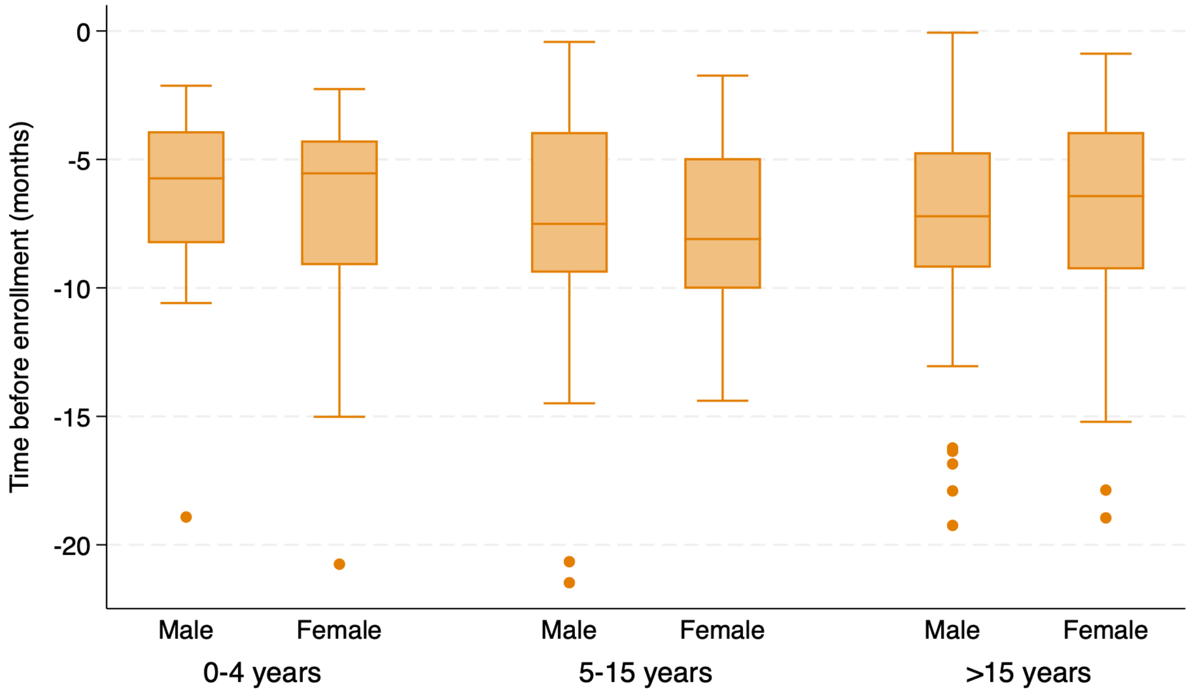

In this figure, the boxes show the interquartile range (IQR), whiskers show the range, and the circles are the outliers. The vivax malaria history episode was detected during patient consultation in the outpatient clinics.

Supplement Figure 3. Comparing contemporary (after primaquine) and historical (without primaquine) annual incidence of *Plasmodium vivax* malaria stratified by age group

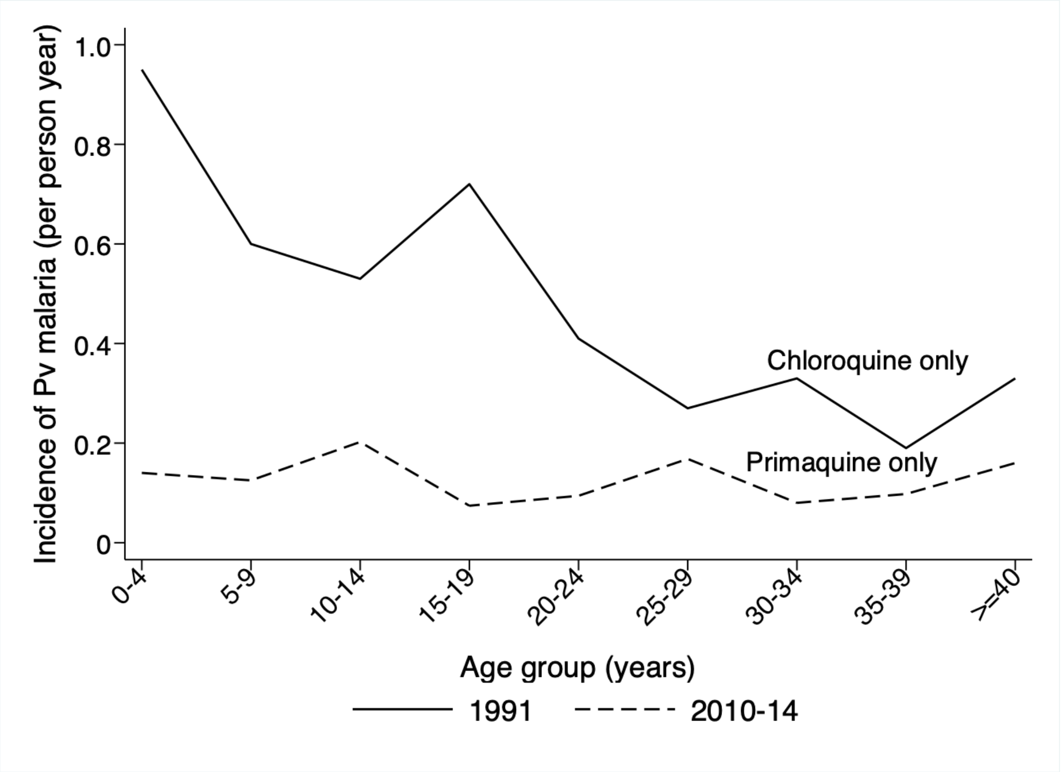

Legend: The annual incidence of *P. vivax* recurrence after radical cure in the current study (2010 to 2014) is compared to the historical incidence rates in 1991, derived from Figure 3 in reference 24 after chloroquine treatment when primaquine was not prescribed routinely for radical cure. *P. vivax* malaria includes actively and passively detected cases. The primaquine dose was 7mg base/kg total dose over 14 days.

Supplement Figure 4. Comparing contemporary (after primaquine) and historical (without primaquine) age stratified annual incidence of *Plasmodium vivax* malaria

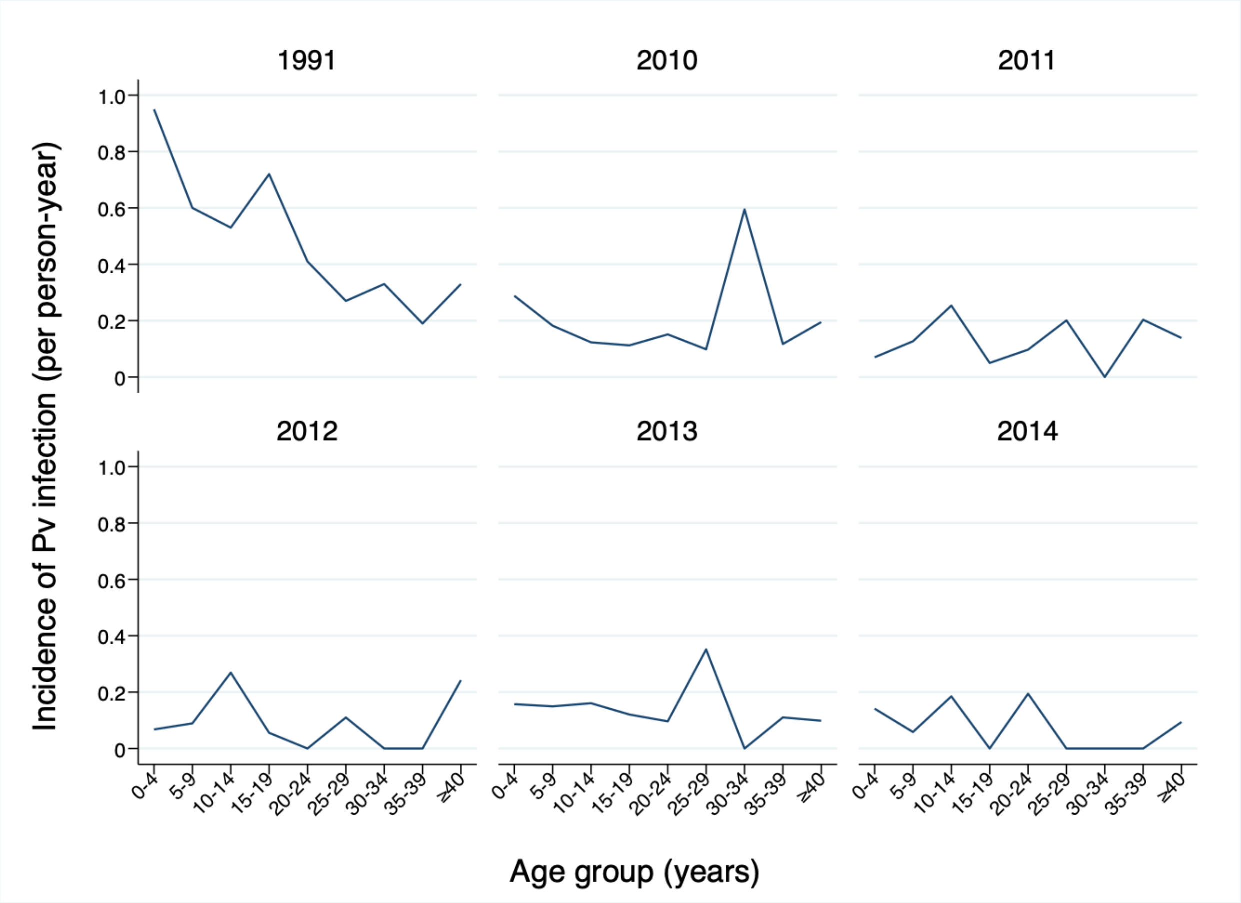

Data from 1991 are from reference 24.

Supplement Figure 5. *Plasmodium vivax* recurrence after enrollment stratified by a history of *Plasmodium vivax* malaria <12 months versus ≥12 months before enrollment

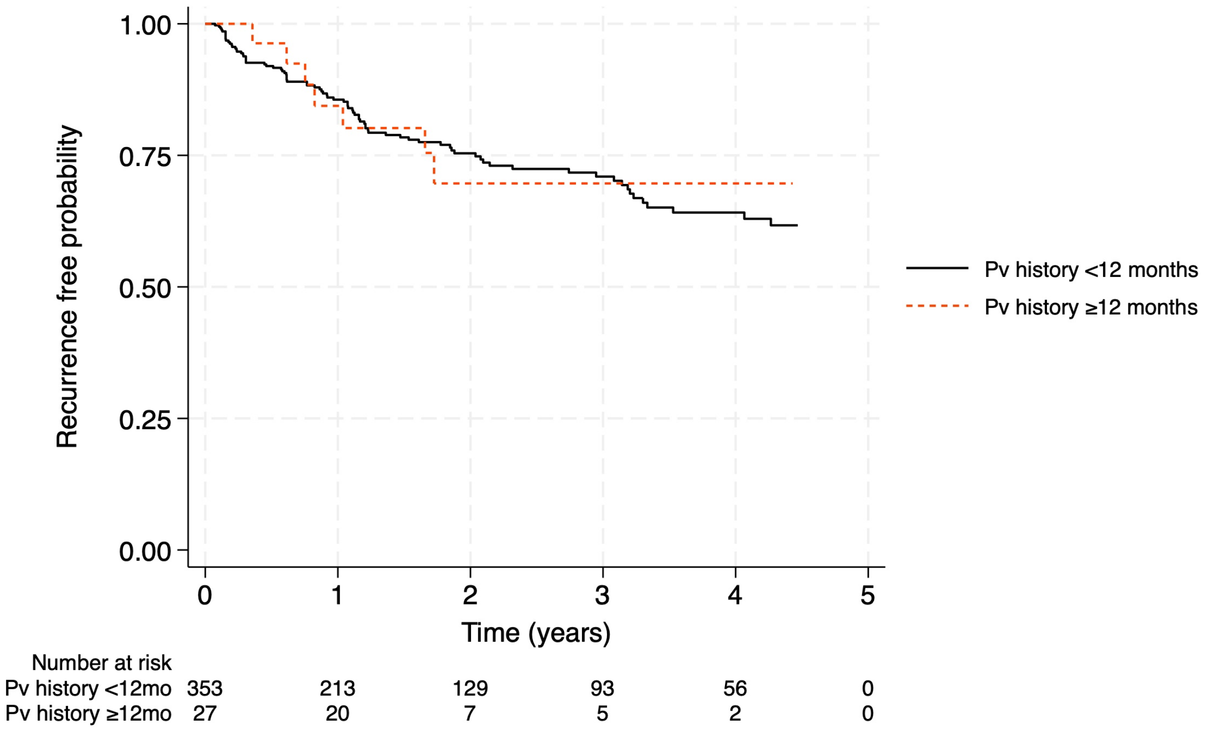

Supplement Figure 6. Village location of participants in this cohort study and two parallel *Plasmodium vivax* chemotherapy trials

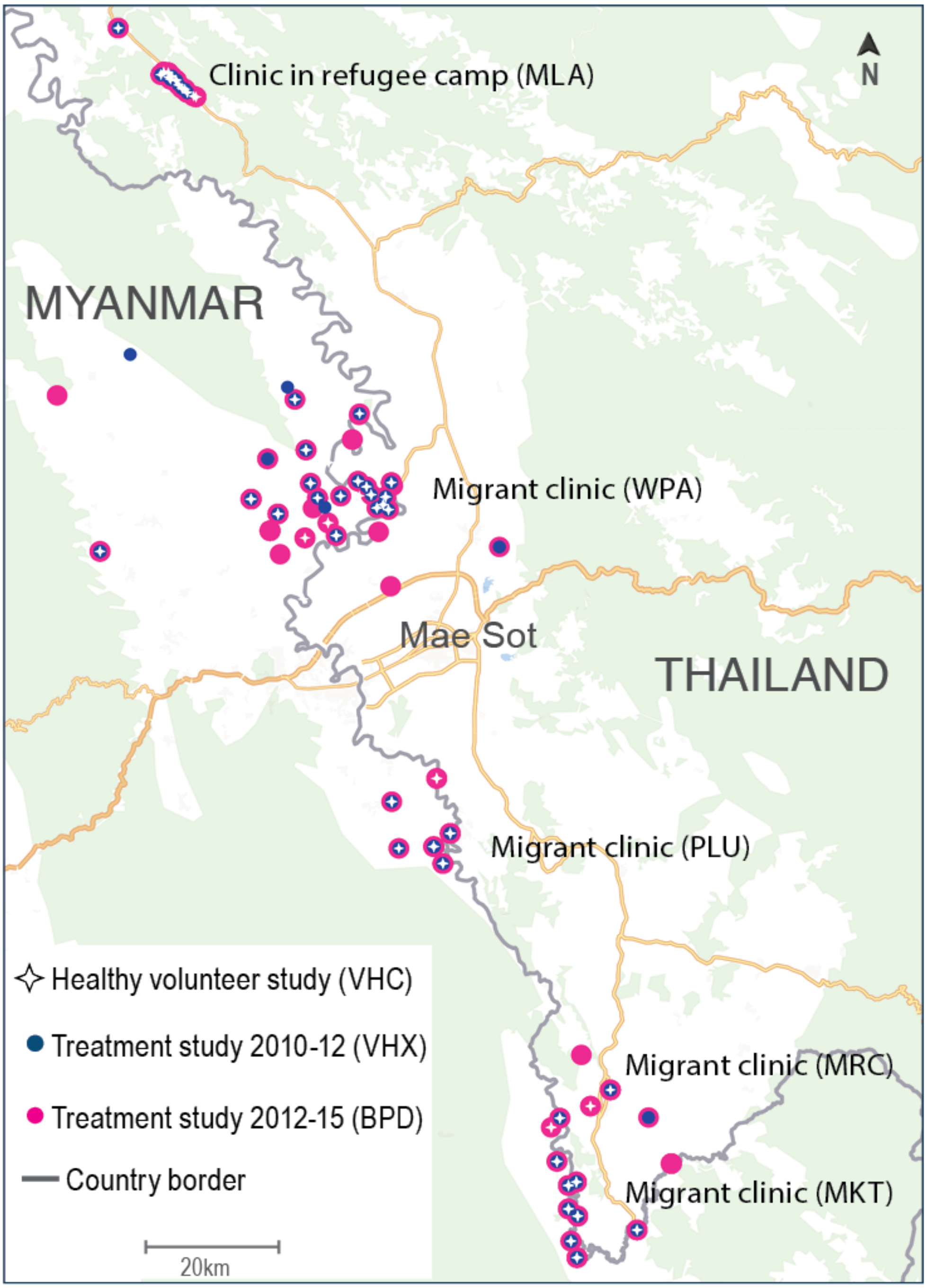

Abbreviations: MKT, Maw Ker Thai; MLA, Maela; MRC, Murunchai; PLU, Mae Khon Khen; Pv, *Plasmodium vivax*; WPA, Wangpha
Plotted data are from the primaquine treatment arms of the VHX and BPD trials (references 8 and 27).
This map was created using OpenStreetMap® licensed under the Open Data Commons Open Database License (ODbL) by the OpenStreetMap Foundation and from © OpenStreetMap contributors (https://www.openstreetmap.org/copyright).
